# Adsorptive Leukocytapheresis in Inflammatory Bowel Diseases: Our Preliminary Results are Encouraging

**DOI:** 10.4274/Tjh.2012.0186

**Published:** 2013-06-05

**Authors:** Murat Törüner, Selami Koçak Toprak, Kiraz Mızrak, Yasin Yıldırım, Meltem Bay, Erol Ayyıldız, Osman İlhan

**Affiliations:** 1 Ankara University, School of Medicine, Department of Gastroenterology, Ankara, Turkey; 2 Başkent University, School of Medicine, Department of Hematology, Ankara, Turkey; 3 Ankara University, School of Medicine, Department of Hematology, Therapeutic Apheresis Unit, Ankara, Turkey

## TO THE EDITOR

Inflammatory bowel disease (IBD), consisting of ulcerative colitis (UC) and Crohn’s disease (CD), is a chronic recurrent disorder with unclear etiology. A close relation to autoimmune status featured by antibodies against colonic epithelial cells was suggested in light of recent studies [1]. Although 5-aminosalicylates and corticosteroids are frequently used in treatment, the management of both conditions is far from being fully satisfactory [2]. Thus, many biological treatment methods, like targeting cytokines involved in intestinal inflammation, have been developed in the last decade with various results in terms of efficacy and safety. One of them is cytapheresis, which aims to suppress and reduce impaired immune responses in the diseased intestine by removing circulating activated leukocytes, especially granulocytes, which have been shown to cause intestinal crypt abscess. 

Two patients (Table 1) who were refractory to their present treatments were administered adsorptive cytapheresis (leukocytapheresis - Cellsorba®) at our unit in 80-min sessions once a week for a total of 5 weeks following required legal permissions. No complications related to the method were observed and in the CD case, the discharge from the fistula stopped, while in the UC case, bloody defecation and the need for erythrocyte suspensions disappeared. In both patients, the quality of life increased along with weight gain and the complaints were almost completely removed.

Leukocytapheresis is an extracorporeal removal of activated granulocytes and monocytes, representing the major source of pro-inflammatory cytokines in the intestinal mucosa, from the blood using special filters or columns. Cellsorba® (Asahi Medical, Tokyo, Japan), an adsorptive column, is made of non-woven polyester fiber filter and is able to remove about 90%–100% of granulocytes and monocytes, 30%–60% of lymphocytes, and 30% of platelets from the peripheral blood [2]. Pro-inflammatory cytokines like TNF-α, IL-2, IL-8, and IFN-γ, which are especially high in patients with IBD, and acute phase reactants like C reactive protein and erythrocyte sedimentation rate are close to the normal limits following leukocytapheresis. Additionally, CD14(dull)CD16+ monocytes, which are specifically known as the source of TNF-α and IL-12, are selectively removed from peripheral blood and, thus, Cellsorba may be presented as an extracorporeal anti-TNF-α therapy [3]. In a pilot study including 20 UC cases refractory to conventional treatments, the procedure was performed once a week for 5 consecutive weeks and a clinical response was observed in 14 patients, while an endoscopic response was observed in 6 patients. In the responding group, the significance of the response in patients receiving monthly maintenance apheresis therapy continued when compared to the observation arm [4]. In another study containing 18 active CD cases, the procedure was performed once a week for 5 consecutive weeks, followed by monthly maintenance apheresis therapy, and it was reported that clinical remission was achieved at a rate of 50% [5]. In our 2 cases, although pro-inflammatory cytokines were not evaluated, the decrease in levels of acute phase reactants and the marked clinical-endoscopic response were both deemed significant (Table 2). However, for leukocytapheresis to show its efficiency and take its place among the first line of treatment choices in IBD and especially CD, further randomized and controlled studies performed on large patient series and including long-term follow-up results are required.

## Figures and Tables

**Table 1:**
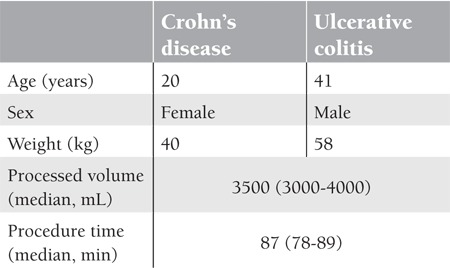
Features of patients and the procedure

**Table 2:**
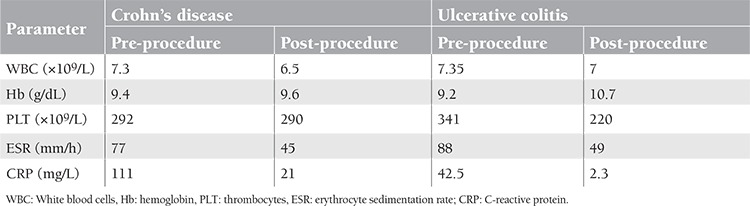
Pre- and post-procedure laboratory tests results.
